# Online Search Interest in Gynecologists After the Release of the Film *Barbie*

**DOI:** 10.1001/jamanetworkopen.2024.24658

**Published:** 2024-07-25

**Authors:** Eva Senechal, Charles F. Bray, Christopher M. Worsham, Anupam B. Jena

**Affiliations:** 1 Department of Experimental Medicine, Faculty of Medicine and Health Sciences, McGill University, Montreal, Quebec, Canada; 2 Department of Health Care Policy, Harvard Medical School, Boston, Massachusetts; 3 Department of Medicine, Massachusetts General Hospital, Boston; 4 Division of Pulmonary and Critical Care Medicine, Massachusetts General Hospital, Boston; 5 National Bureau of Economic Research, Cambridge, Massachusetts

## Abstract

This cross-sectional study evaluates whether the film *Barbie* was associated with increased public interest in gynecologic care in the US after its release.

## Introduction

Popular culture has been shown to influence health behaviors among the general public.^1^ For example, Katie Couric’s live streamed colonoscopy was associated with a transient 21% increase in colonoscopies, Angelina Jolie’s essay about her experience with breast cancer led to a transient 64% increase in genetic testing, and a 29% increase in suicide rates was observed in the months following the controversial season finale of *13 Reasons Why*.^2,3,4^ The movie *Barbie* was released July 21, 2023, and sold 12.8 million tickets during its debut weekend, becoming one of the highest grossing films ever.^5^ While comedic, *Barbie* addresses serious topics about womanhood. In the film’s final scene, after deciding to leave Barbieland for the real world, Barbie enthusiastically tells a receptionist, “I’m here to see my gynecologist,” a joke that could be based either on her supposed lack of genitals or her evident excitement for care many women find unpleasant. We hypothesized that this final line may have spurred public interest in gynecologic care.

## Methods

In this cross-sectional study, we analyzed online search trends in the US following *Barbie*’s release, focusing on a list of 34 queries related to understanding or seeking gynecologic care, women’s health care, or medical care more broadly (Figure). This study was deemed not human participant research by the Harvard Medical School Institutional Review Board and was therefore exempt from approval and the need for informed consent. The study followed the STROBE reporting guideline.

**Figure.**
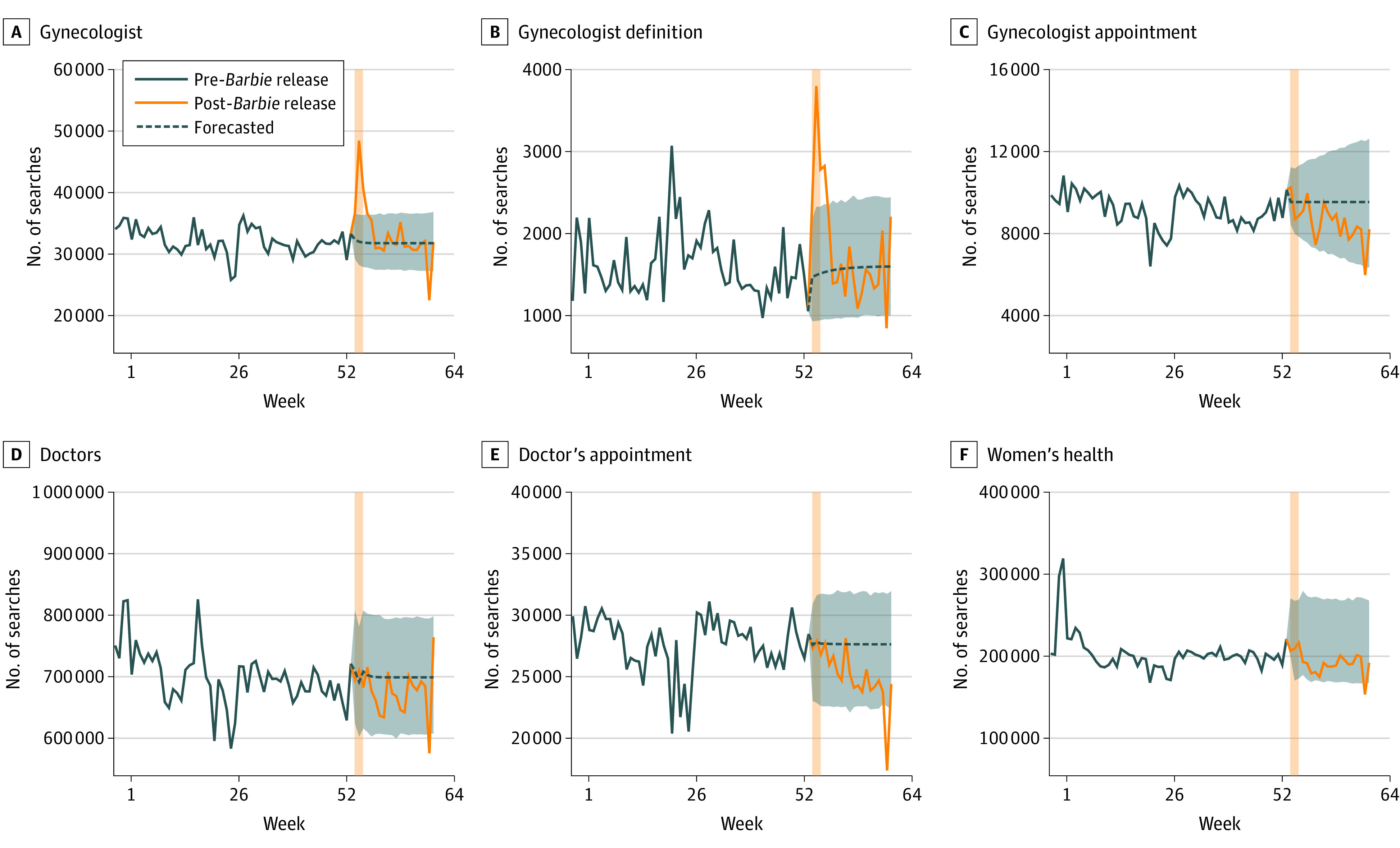
Search Trends for Gynecology and Related Terms Following the Release of the Film *Barbie* The y-axis represents the aggregate weekly US search volume for search terms within the category. Vertical bars coincide with *Barbie*’s release date of July 21, 2023. Categories represent aggregate searches for the following terms (alternate spellings, eg, “gynaecologist,” were also included but are not listed for brevity): gynecologist = {“gynecologist,” “gynecology”}; gynecologist definition = {“gynecologist definition,” “gynecologist meaning,” “what is a gynecologist,” “what does a gynecologist do,” “why see a gynecologist,” “do I need a gynecologist”}; gynecologist appointment = {“gynecologist appointment,” “gynecologist near me,” “find a gynecologist”}; women’s health = {“womens health,” “preventive health care for women,” “reproductive health,” “birth control,” “contraceptives,” “breast health,” “mammogram,” “pap smear”}; doctors = {“doctor,” “primary care physician,” “family doctor,” “general practitioner,” “pediatrician,” “cardiologist,” “oncologist”}; doctor’s appointment = {“doctors appointment,” “doctors near me,” “find a doctor,” “make a doctors appointment”}.

Search queries were grouped into several categories: gynecologist, gynecologist definition, gynecologist appointment, women’s health, doctor, and doctor’s appointment. The first 3 categories measured topics directly related to the language used in *Barbie*. The last 3 categories assessed whether unobserved contemporaneous factors influencing health-seeking behavior more generally may have contributed to gynecologic-related search volume and, thus, served as control search terms. Weekly US online search trends in the year preceding and 3 months following the July 21, 2023, release date were obtained using Google Trends and Glimpse. Other than *Barbie*’s release, there were no major events during this period that may have led to widespread changes in search interest in gynecology.

Observed search volumes following *Barbie*’s release were compared with predicted search volumes generated using autoregressive integrated moving average based on weekly search data from June 2, 2022, through the week before the release. Percentage differences between measured and predicted search volumes were calculated as simple ratios, with 95% CIs calculated based on bootstrapped standard errors. The data analysis was performed using R, version 4.3.3 (R Foundation for Statistical Computing). A 2-sided *P* < .05, Bonferroni corrected for multiple outcomes, was considered significant.

## Results

In the week following *Barbie*’s release, there were large increases in the national online search volume for terms referring to gynecologists (51.3%; 95% CI, 31.8%-72.1%; *P* < .001) and gynecologist definition (154.1%; 95% CI, 68.2%-304.5%; adjusted *P* = .03) (Figure). Meanwhile, there were no changes in searches for gynecologist appointments, suggesting that searches for information about gynecologists did not translate to searches for new gynecologic care. No changes were observed for search terms reflecting broader health interest (eg, searches for doctors’ appointments), supporting the assumption that the observed increase in gynecologist-related searches may have been influenced by the film’s release and not other factors.

## Discussion

Our results suggest that *Barbie*’s closing line may have spurred interest in gynecology, further suggesting the potential influence of popular films on health literacy and awareness. While there were no changes in search volume associated with seeking care, a primary limitation of the study is that such changes in behavior may not be adequately captured by search trends, and in particular, they may be temporally far removed from changes in awareness. Furthermore, individuals searching for information about gynecologists may not themselves require gynecologic care. Therefore, it remains unclear whether a “*Barbie* effect” in awareness would translate to improved measurable health outcomes.

## Notes

Supplement.
Data Sharing Statement
