# Supplementary material for: Online Search Interest in Gynecologists After the Release of the Film Barbie
Source: JAMA Netw Open. 2024 Jul 25;7(7):e2424658. doi: 10.1001/jamanetworkopen.2024.24658 (PMC11273229; doi:10.1001/jamanetworkopen.2024.24658)
Supplement: Supplement. — Data Sharing Statement [file jamanetwopen-e2424658-s001.pdf]

Data Sharing Statement

Senechal. Online Search Interest in Gynecologists After the Release of the Film Barbie. *JAMA Netw Open*. Published July 25, 2024. doi:10.1001/jamanetworkopen.2024.24658

Data
Data available: No
